# Ground Beef Recall Associated with Non-O157 Shiga Toxin–producing *Escherichia coli*, United States

**DOI:** 10.3201/eid2001.130915

**Published:** 2014-01

**Authors:** Amy Robbins, Madhu Anand, David C. Nicholas, Jessica S. Egan, Kimberlee A. Musser, Steve Giguere, Hal Prince, Henrietta E. Beaufait, Stephen D. Sears, James Borda, Debbie Dietz, Thomas Collaro, Peter Evans, Scott A. Seys, Bonnie W. Kissler

**Affiliations:** Maine Department of Health and Human Services, Augusta, Maine, USA (A. Robbins, S.D. Sears);; New York State Department of Health, Albany, New York, USA (M. Anand, D.C. Nicholas, J.S. Egan, K.A. Musser);; Maine Department of Agriculture, Augusta (S. Giguere, H. Prince, H.E. Beaufait);; United States Department of Agriculture, Philadelphia, Pennsylvania, USA (J. Borda);; United States Department of Agriculture, Pittsburgh, Pennsylvania, USA (D. Dietz);; United States Department of Agriculture, Waltham, Massachusetts, USA (T. Collaro);; United States Department of Agriculture, Washington, DC, USA (P. Evans);; United States Department of Agriculture, Minneapolis, Minnesota, USA (S.A. Seys);; United States Department of Agriculture, Atlanta, Georgia, USA (B.W. Kissler)

**Keywords:** Shiga toxin–producing Escherichia coli, STEC, foodborne illness, foodborne disease, ground beef, traceback, Maine, New York, bacteria

**To the Editor:** Shiga toxin–producing *Escherichia coli* (STEC) cause severe illness in humans, especially young and elderly persons. In previous decades, prevention and control measures focused on STEC O157:H7; however, in recent years, non-O157 STEC–related outbreaks and illnesses have been detected more frequently. In the United States, 6 serogroups (O26, O45, O103, O111, O121, and O145) account for ≈75% of the reported non-O157 STEC illnesses (*1*).

On August 4, 2010, the Maine Center for Disease Control and Prevention (Maine CDC) investigated 2 isolates of nonmotile STEC O26 that were indistinguishable by pulsed-field gel electrophoresis (PFGE). Both case-patients had diarrhea and abdominal cramps, shopped at grocery stores in the same town, and reported consumption of ground beef. Case-patient 1 purchased ground beef at Store A; a shopper card used for the purchase was shared with investigators. Case-patient 2 consumed ground beef purchased from 2 stores (Stores B and C); neither shopper cards nor receipts were available.

On August 5, a Maine Department of Agriculture, Food and Rural Resources (Maine DoA) inspector visited Stores A and B. On June 25, case-patient 1 had purchased 90% lean ground beef at Store A; the beef was produced by a parent company with multiple establishments. Inspectors cross-referenced this purchase with meat grinding logs from Store B, which revealed that the parent company that supplied ground beef to Store A also supplied beef to Store B. Maine DoA notified the United States Department of Agriculture, Food Safety and Inspection Service (USDA-FSIS), of a common manufacturer.

On August 9, the New York State (NYS) Department of Health contacted Maine CDC regarding a third case-patient with an STEC O26 isolate that was indistinguishable by PFGE from the other 2 isolates. Case-patient 3 had handled 90% lean ground beef purchased from the grocery store chain used by case-patient 1 (Store A). Shopper card information indicated that the beef was purchased on June 17. Ground beef was the only common exposure among the 3 case-patients.

During August 18–26, Maine DoA, NYS Department of Agriculture and Markets, and USDA-FSIS conducted a traceback of ground beef (Figure). Traceback revealed that for >10 years, Store A had been purchasing 90% lean ground beef from Establishment X (1 of many establishments within the parent company). Further investigation revealed that implicated ground beef from Store A locations in Maine and New York had come from the same lot at Establishment X. USDA-FSIS conducted ground beef traceback at Stores B and C; source materials were received from multiple establishments, but Establishment X was the only common supplier (Figure). On August 28, Establishment X recalled ≈8,500 pounds of ground beef that had been produced on June 11.

**Figure:**
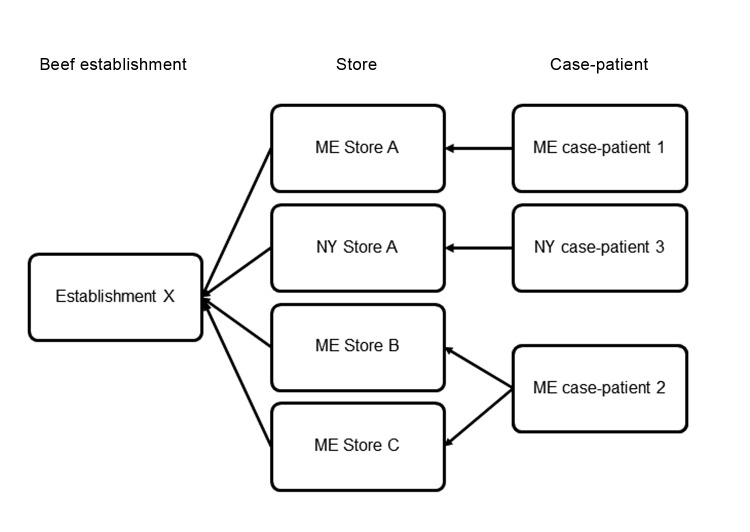
Supplier traceback for ground beef associated with a cluster of *Escherichia coli* O26 infections in Maine (ME) and New York (NY), 2010.

On September 2, the NYS Department of Health Public Health Laboratory tested leftover hamburger patties purchased by case-patient 3. The samples were confirmed as STEC O26 with a PFGE pattern indistinguishable from the strains isolated from case-patients.

On November 17, USDA-FSIS completed an assessment at Establishment X and determined that the company’s food safety system was adequate to control pathogens of concern. Follow-up testing of beef trim samples at Establishment X were negative for STEC O26 and O157:H7.

The Council to Improve Foodborne Outbreak Response guidelines emphasize the importance of timely involvement of all members in outbreak investigations (*2*). A review of enteric disease investigations by Hedberg et al. (*3*) concluded a need to increase timeliness of case investigation and to reduce delays during outbreak investigations. While waiting for PFGE confirmation, Maine CDC notified Maine DoA of case-patients who purchased ground beef in the same city. Within 48 hours, an inspector visited grocery stores where case-patients purchased ground beef and notified USDA-FSIS of the illness investigation. Ten days after the shopper card information from case-patient 3 was available, USDA-FSIS convened a recall committee. Quick action by all agencies led to timely investigation, traceback, and recall. This well-characterized outbreak of only 3 cases of STEC O26 infection led to a recall of ground beef.

Foodborne illness investigations increasingly rely on purchase records from shopper cards, which record information such as purchase dates, brands, and product types that are valuable for traceback and identification of common exposure to a food item. After a thorough record review, investigators in this outbreak were able to narrow the purchased beef to 1 production date. This finding emphasizes the importance of recordkeeping at retail stores and meat processing establishments to determine production dates in the event of a recall. Shopper cards are used more frequently during investigations, so safeguards to protect the consumers’ personally identifiable information are needed to prevent inappropriate disclosure and accidental breeches of confidentiality.

In an effort to reduce human illnesses, USDA-FSIS developed policy on non-O157 STEC in raw beef products to declare 6 serogroups of pathogenic STEC as adulterants in nonintact raw beef (*4*). The Agency began implementing routine testing for these serogroups in June 2012. 
